# Feasibility of mitigation measures for agricultural greenhouse gas emissions in the UK. A systematic review

**DOI:** 10.1007/s13593-023-00938-0

**Published:** 2023-12-28

**Authors:** Asma Jebari, Fabiana Pereyra-Goday, Atul Kumar, Adrian L. Collins, M. Jordana Rivero, Graham A. McAuliffe

**Affiliations:** 1 Net Zero and Resilient Farming, Rothamsted Research, North Wyke, Okehampton, EX20 2SB Devon UK; 2 Instituto Nacional de Investigacion Agropecuaria (INIA), Ruta 8 km 281, Treinta y Tres, postcode 33000 Montevideo, Uruguay

**Keywords:** Net zero, Carbon footprint, Farming interventions, Arable farming, Livestock systems, Mixed farming

## Abstract

The UK Government has set an ambitious target of achieving a national “net-zero” greenhouse gas economy by 2050. Agriculture is arguably placed at the heart of achieving net zero, as it plays a unique role as both a producer of GHG emissions and a sector that has the capacity via land use to capture carbon (C) when managed appropriately, thus reducing the concentration of carbon dioxide (CO_2_) in the atmosphere. Agriculture’s importance, particularly in a UK-specific perspective, which is also applicable to many other temperate climate nations globally, is that the majority of land use nationwide is allocated to farming. Here, we present a systematic review based on peer-reviewed literature and relevant “grey” reports to address the question “how can the agricultural sector in the UK reduce, or offset, its direct agricultural emissions at the farm level?” We considered the implications of mitigation measures in terms of food security and import reliance, energy, environmental degradation, and value for money. We identified 52 relevant studies covering major foods produced and consumed in the UK. Our findings indicate that many mitigation measures can indeed contribute to net zero through GHG emissions reduction, offsetting, and bioenergy production, pending their uptake by farmers. While the environmental impacts of mitigation measures were covered well within the reviewed literature, corresponding implications regarding energy, food security, and farmer attitudes towards adoption received scant attention. We also provide an open-access, informative, and comprehensive dataset for agri-environment stakeholders and policymakers to identify the most promising mitigation measures. This research is of critical value to researchers, land managers, and policymakers as an interim guideline resource while more quantitative evidence becomes available through the ongoing lab-, field-, and farm-scale trials which will improve the reliability of agricultural sustainability modelling in the future.

**Supplementary Information:**

The online version contains supplementary material available at 10.1007/s13593-023-00938-0.

**Contents**

- 1. Introduction
- 2. Structure of the systematic review
  - 2.1 Search strategy
  - 2.2 Publication databases
  - 2.3 Article screening and study eligibility criteria
  - 2.4 Study validity assessment
  - 2.5 Data extraction strategy
  - 2.6 Data synthesis and presentation
- 3. Mitigation measures
  - 3.1 Arable systems’ mitigation measures
  - 3.2 Livestock systems
    - 3.2.1 Manure management
    - 3.2.2 Grassland management: fertilization and extensification
  - 3.3 Livestock diets
    - 3.3.1 Supplements to inhibit greenhouse gas production
    - 3.3.2 Modifying feeding regimes
  - 3.4 Livestock health and genetic performance
  - 3.5 Horticultural systems on peatlands
  - 3.6 Mixed farm systems and their role in sustainable agriculture
  - 3.7 Offsetting greenhouse gas emissions on agricultural land
  - 3.8 Bioenergy production
- 4. Limitations and critical gaps for future research
  - 4.1 Limitations
  - 4.2 Critical gaps for future research
- 5. Conclusions
- References

## Introduction

While agriculture contributes less than 1% to the United Kingdom’s (UK’s) economy, it provides around three-quarters of domestic food consumption and utilizes around 71% of the land. Approximately 72% of the latter is used for grazing systems and 26% for arable crops including cereals, oilseeds, and potatoes, with the remaining land (~2%) being utilized for produce such as medicinal plants and herbs (Defra 2021). As a food-trading nation, the UK relies on both imports and a thriving domestic agricultural sector to feed itself and drive economic growth (ADAS 2019). In the most recent national inventory assessment of UK emissions, agriculture accounted for ~10% of total greenhouse gas (GHG) emissions (Brown et al. 2020). Despite the relatively low total emissions arising from primary food production compared to other sectors, such as energy and transport (BEIS 2022), the agricultural sector is the major source of both nitrous oxide (N_2_O) and methane (CH_4_) emissions in the UK, both of which are powerful and complex GHGs, accounting for nearly 69% of total N_2_O emissions and 48% of total CH_4_ emissions in the UK, respectively (Defra 2021). In contrast, agriculture only accounts for ~1.7% of total carbon dioxide (CO_2_) emissions (Defra 2021). More specifically, nearly 90% of agricultural N_2_O emissions originate from soils through microbial (de)nitrification of nitrogen-based fertilizers, farmyard manure (FYM), and deposition of urine and feces on grazing/foraging lands and indirectly through leaching/runoff and volatilization primarily from ammonia (NH_3_). Most CH_4_ emissions (~90%) arise from enteric fermentation (digestive processes, specifically eructation) in ruminant animals, with manure management practices accounting for the remainder.

The agricultural sector accounted for 88% of the UK’s NH_3_ emissions in 2021 (Defra 2021). NH_3_ is generated from the application of synthetic (e.g., ammonium nitrate) and organic fertilizers (e.g., slurry and manure) to soils and during storage. Further, while rates of soil erosion in England are not excessively high by global standards, rates on agricultural land are elevated relative to those under natural land covers, resulting in elevated sediment delivery to rivers (Collins and Zhang 2016; Collins et al. 2021) leading to off-farm impacts including degradation of aquatic ecology (e.g., Kemp et al. 2011) and the siltation of drinking water reservoirs (Foster et al. 2011).

**Fig. 1:**
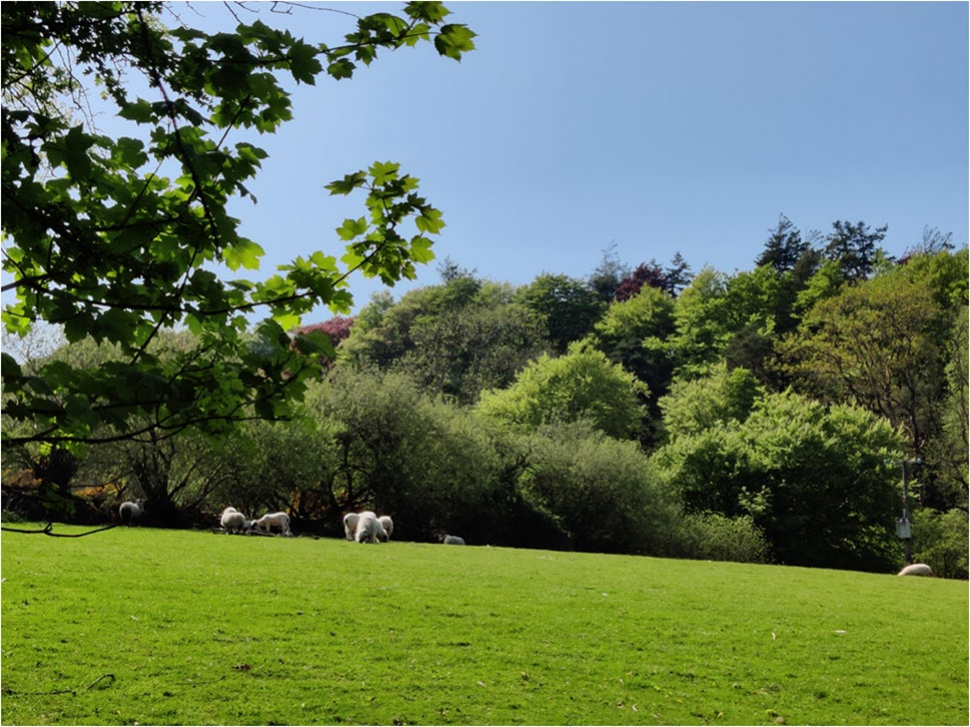
Example of offsetting mitigation measure: planting hedgerows into sheep-grazed pasture in southwest England. Apart from GHG mitigation potential, through SOC sequestration, establishing hedgerows provides a range of co-benefits to livestock and the landscape. Trees can boost production, improve animal health and welfare, and provide wider environmental benefits (see Section 3.7).

The Committee on Climate Change (CCC) has recommended a 64% reduction in GHG emissions from the agriculture and land use sector to meet the national 2050 net-zero GHG target in the UK (CCC 2020). The fact that this is not a 100% reduction reflects the natural biological baseline emissions associated with primary food production (e.g., even if the land was “rewilded,” there would still be baseline emissions arising from unproductive land, due to microbial activity during natural decomposition cycles) (CIEL 2020). In line with the CCC, the National Farmers Union (NFU) of England and Wales established an ambitious goal of net zero by 2040, while assuring climate-friendly food production with high standards of food safety, animal welfare, and environmental stewardship. For instance, agriculture will need to reduce emissions from its production and increase its potential to sequester soil organic carbon (SOC) through land occupation optimization, with GHG offsetting strategies (Fig. 1) such as afforestation and silvopastoral systems being prime exemplars of mitigation pathways (Eory et al. 2020).

Mitigation measures for delivering the UK Government’s net-zero target by 2050 must consider both the economic (e.g., food production and reliance on imports) and environmental sustainability of production systems going forward (CIEL 2022). Furthermore, the NFU highlighted the fact that the transition of agriculture to net-zero GHGs must ensure the economic, environmental, and social benefits of farming, such as supporting rural workforces and delivery of nutritious produce, are protected (NFU 2021a). Environmental scientists and engineers, social scientists, nutritional scientists, and economists are therefore tasked to seek ways to increase productivity while at the same time reducing environmental damage and maintaining the healthy function of agroecosystems (e.g., increasing biodiversity, often measured as species losses-gains per year, while simultaneously reducing GHGs) in the long term (Tilman et al. 2011; Tilman and Clark 2014).

An important part of working towards net zero includes the accurate accounting of GHG emissions. The national inventory accounting forms the basis of international climate change treaties (e.g., the Kyoto Protocol). Another, more holistic, approach to quantifying supply-chain-level environmental impacts is life cycle assessment (LCA), a deterministic modelling framework widely used in agricultural sustainability analyses. In contrast to national GHG inventories, detailed LCAs quantify losses of pollutants occurring in other countries for imported products associated with food production, such as animal feed (e.g., displaced protein-sources imported from the Americas), and fertilizer chemicals (CIEL 2020). As such, LCA provides a deeper, global view of the C footprint for any product or service (Müller et al. 2020). However, despite recent computational and mathematical improvements to LCA, data availability remains one of the major limiting factors when utilizing the framework to answer pressing societal concerns pertaining to environmental degradation (McAuliffe 2020a). In the absence of suitable life cycle inventory analyses (LCI) material flows, assessing the potential of GHG abatement efforts is challenging. For instance, predictions made by scenario-based LCA models in the context of net zero are currently liable to high degrees of uncertainty, despite numerous methodological capabilities to capture such data-based restrictions (ISO 2006; Cain et al. 2019; Müller et al. 2020; McAuliffe et al. 2020b).

Systematic reviews provide a rigorous, objective, and transparent means of creating a searchable database of relevant academic and grey literature (Kohl et al. 2018), while providing an opportunity to clarify the current evidence base and highlight important knowledge gaps. To the best of our knowledge, the most recent review on climate change mitigation in the UK was a literature review that focused only on cropping systems (i.e., food-crop production, particularly arable systems including root crops; Rial-Lovera et al. 2017). Other reviews related to broader sustainability assessments (e.g., exploration of environmental impacts including water pollution and terrestrial acidification, both of which indirectly produce GHGs and thus affect the achievement of net zero) have covered livestock in general (de Vries and de Boer 2010), beef production (de Vries et al. 2015), pig production (McAuliffe et al. 2016), the nutrition-environment nexus (McAuliffe et al. 2020a), and technical issues related to complexities such as how to allocate burdens arising from dairy systems which produce multiple (co)products such as milk and beef (Rice et al. 2017).

In this new systematic review, we synthesized a quantitative *and* qualitative dataset (see data in brief in Jebari et al. (2023)) of existing and potentially viable GHG mitigation measures and technologies which can be deployed on farms, regardless of whether they are arable, livestock, or mixed farms, including rotational systems. We refer to scientific literature and aggregated data that are key to the net-zero objectives, thus exploring environmental, economic, and societal perspectives for different mitigation measures.

## Structure of the systematic review

### Search strategy

**Fig. 2:**
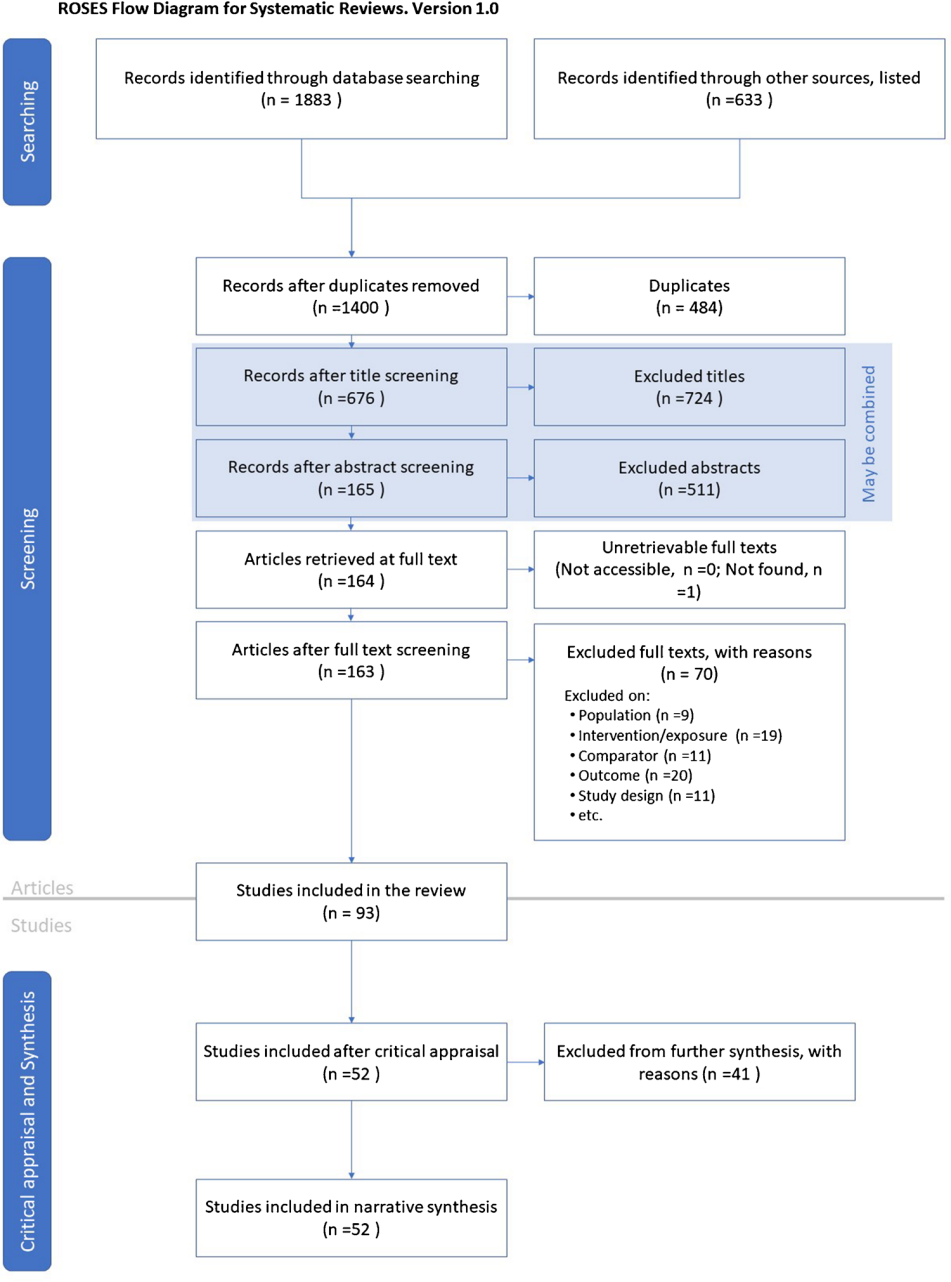
RepOrting standards for Systematic Evidence Syntheses (ROSES) flow diagram (Haddaway et al. 2018) showing literature sources and inclusion/exclusion process.

We followed the Collaboration for Environmental Evidence (CEE) guidelines and methodology therein to create our systematic review (CEE 2018) (Fig. 2). Only papers or reports published in English were considered for inclusion under the following structure:

- **Activity terms**: “arable crops,” “cereal,” “wheat,” “barley,” “oilseed,” “potato,” “horticulture,” “livestock,” “dairy,” “beef,” “cattle,” “pig,” “sheep,” “poultry,” “chicken,” “turkey,” “mixed farm,” “cow,” “grassland,” “pasture,” “oat”
- **Intervention terms**: “management,” “practice measures,” “alternative technology”
- **Outcome terms**: “carbon footprint,” “greenhouse gas emissions,” “direct emissions,” “indirect emissions,” “methane,” “nitrous oxide,” “carbon dioxide,” “ammonia,” and “nitrate.”

The search terms within each of the three categories (activity, intervention, and outcome) were combined using the Boolean operator “OR.” We combined the three categories into a search string using the Boolean operator “AND.” The search string was modified depending on the functionality of different databases (e.g., looking for keywords or topics), specialist sustainability-related websites, and search engines (e.g., *Scopus*). The temporal boundary of the literature search applied included recent relevant information and data published during the last 5 years (i.e., between 2017 and 2022). The purpose was to update the most recent literature and available technological advances in the agricultural sector of the UK. All the searches were performed in English in June 2022. The geographic boundary focused as far as feasibly possible on UK-specific literature; however, studies which covered multiple nations, including the UK, were also assessed. Despite focusing primarily on the aforementioned temporal boundary, older material sourced via “snowball” searching (i.e., identifying relevant sources of information via reference lists within the retrieved papers and reports) was also assessed to target novel, updated research streams. Recorded references were imported into *Mendeley* library and *Rayan* (online systematic review software) (Ouzzani et al. 2016). All duplicates were removed, and their numbers were recorded (Jebari et al. 2023).

### Publication databases

The search included the following online scientific databases:

1. Web of Science Core Collection (https://mjl.clarivate.com/home)
2. Scopus (https://www.scopus.com/)
3. Rothamsted Repository (https://repository.rothamsted.ac.uk/)
4. British Library (ETHOS) (https://ethos.bl.uk/)
5. Formerly American Doctoral Dissertations (EBSCO) (http://search.ebscohost.com/login.aspx?authtype=ip,athens&custid=ns010809&group=main&profile=ehost)

Specialist websites of relevant UK organizations listed below were also searched in June 2022 for links or references to relevant articles and data (i.e., “snowball sampling,” as mentioned previously), including grey literature:

1. Department for Environment and Rural affairs (Defra) (http://defra.gov.uk/)
2. National Farmers' Union (NFU) (https://www.nfuonline.com/)
3. Bangor University (http://www.bangor.ac.uk/)
4. North Wyke Publications Platform (https://www.rothamsted.ac.uk/north-wyke-farm-platform

### Article screening and study eligibility criteria

Article screening was evaluated for relevance based on the eligibility criteria at three levels, title, abstract, and full text, using the systematic review software *Rayan*. Articles were first evaluated for eligibility based on their titles. The primary strategy was to be as inclusive as possible within the boundaries described in Section 2.1. Each article found to be relevant based on its abstract was judged for eligibility by screening the full text. The excluded articles dealt with keywords related to health or food industry (either upstream or downstream from the farmgate), and coastal and marine ecosystems, rather than agricultural systems. Additionally, phosphorus pollution was omitted due to its negligible impacts on GHG emissions (interactions of nutrients within soils and the influence of nutrient ratios, a complex topic, were beyond the scope of the current study). Moreover, experiments conducted outside the UK or under arid or Mediterranean climate conditions were also eliminated.

### Study validity assessment

Eligible studies were subject to a critical appraisal. We assessed study validity and categorized relevant studies as “validated,” “not validated,” and “unclear validity” (the latter could also be considered “inconclusive”). Validity criteria included both susceptibilities to bias (internal validity: study design, strength of evidence, and reliability/replicability) and relevance of the study for our review questions (i.e., external validity). A study was excluded from the narrative synthesis due to internal validity if any of the following factors applied:

1. It does not have replicates (i.e., less than two independent experimental/observational units), in the case of experimental studies.
2. It does not include any uncertainty or sensitivity analysis or assessment of the predicted output against measured data, in the case of modelling studies.

If none of the above factors applied, the study was validated, as it complied with both external and internal validity (as explained above), whereas studies considered to possess unclear validity were subject to internal yet independent revision to judge whether the study is validated or not. A study was categorized to be “unclear” if it did not report sufficient details to judge its validity, for instance, if there is a vague methodological description or if it is difficult to interpret the efficacy of the mitigation measure discussed.

**Fig. 3:**
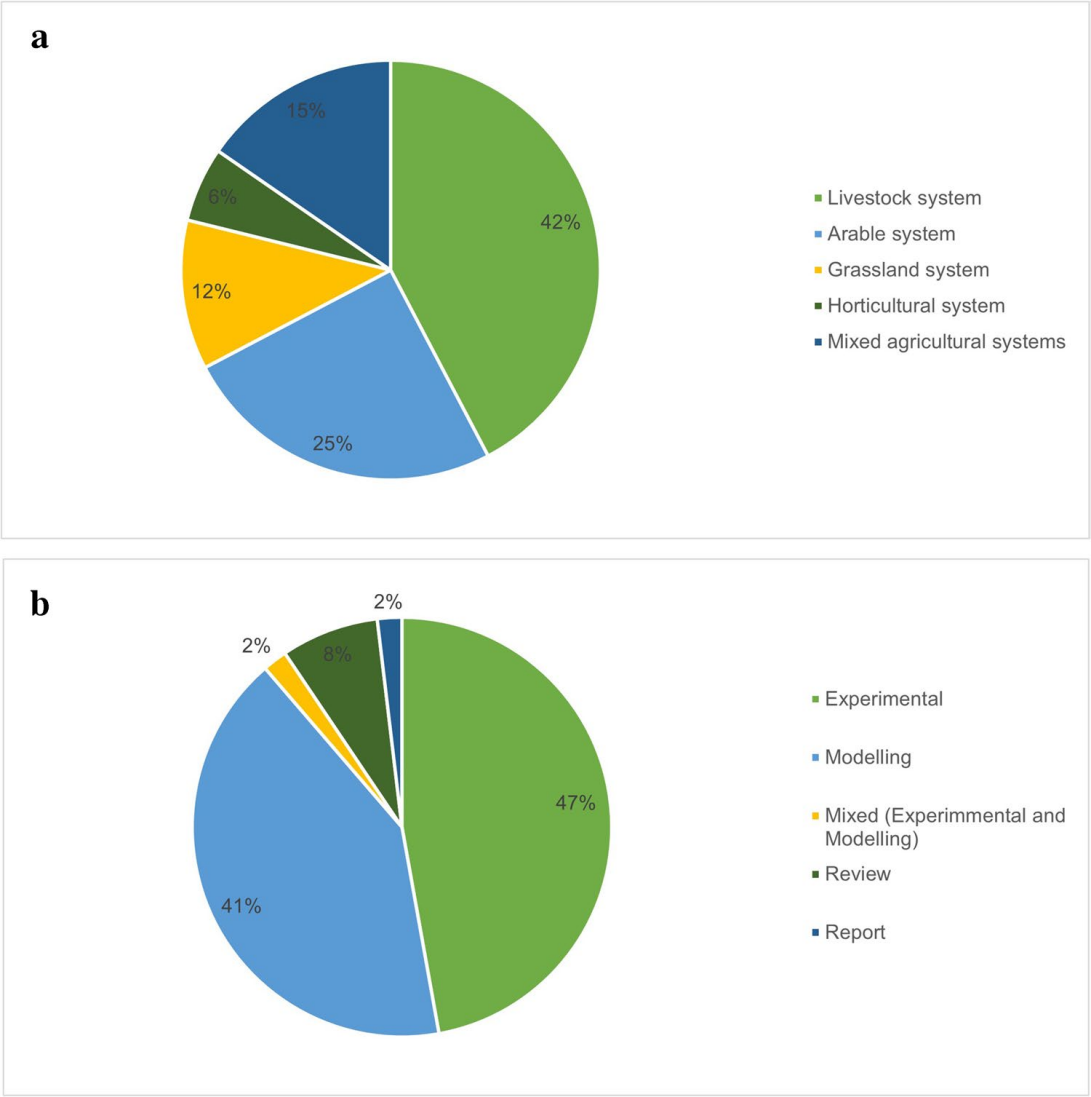
Number of studies per agricultural system (**a**) and study category (**b**).

The final validated studies were included in the narrative synthesis. It is worth noting that we considered different agricultural systems and both modelling and experimental studies (Fig. 3a, b). The final list of included papers, which cover several mitigation measures with various impacts and objectively defined win-win strategies (i.e., reducing GHG emissions while improving agricultural productivity), was reported with recommendations for future research. Studies at the global scale were assessed in terms of the mitigation potential related to the UK.

### Data extraction strategy

We extracted data (and metadata, where applicable) on study characteristics (e.g., whether the study deals with experimental or modelling approaches, or both), description of exposure, outcomes, and study findings. In the case of missing or vague quantitative values pertaining to GHG mitigation measures from the main manuscript, data from available supplementary material, as well as graphs using WebPlotDigitiser (https://automeris.io/WebPlotDigitizer/), were used. We also contacted authors for missing data. All extracted data were quality controlled. Quality control was conducted to identify the value of mitigation and implications of each mitigation measure.

To ensure that the extraction of data and metadata was replicable, entries were subsequently extracted by one author and cross-checked by another author as part of the quality control process. All disagreements amongst team members were discussed and the coding scheme was subsequently adjusted and clarified. Missing data were simply defined as “not stated.”

### Data synthesis and presentation

A qualitative synthesis of a semi-quantitative dataset was conducted as the primary goal to initiate a strategic pathway to net zero through the interpretation of state-of-the-art sustainability literature with a specific focus on GHG mitigation. The coding of the data presented in the synthesis is illustrated in supplementary Table S1 (see Supplementary Material). Our coding process refers to the different questions addressed in the introduction section, regarding the mitigation measure, and its implications in terms of economy, environmental sustainability (particularly GHG emissions), food security, and energy.

## Mitigation measures

The outputs derived from the systematic review are summarized in Tables S1, S2, and S3, according to the primary mitigation measures under three pillars: reducing emissions and/or production efficiency (Table S1), offsetting, and bioenergy production (Table S3).

### Arable systems’ mitigation measures

Regenerative and soil conservation practices, such as cover cropping and reduced tillage, enhance SOC stocks while providing important ecosystem services such as enhancing water retention and reducing soil erosion. As a result, cover cropping with legumes for different arable crops demonstrates sequestration up to 800 kg C ha^−1^ year^−1^ (Glenk et al. 2017) and could potentially sequester up to 16% C up to the year 2050 (Jordon et al. 2022). Similarly, reduced tillage was estimated to sequester up to 100 kg C ha^−1^ year^−1^ (Glenk et al. 2017) and reduce up to 25% of GHG emissions at 5 cm depth in arable cropland (Alskaf 2018). In this context, a global meta-analysis of 946 paired data from 116 peer-reviewed studies showed that, overall, no tillage reduced global warming potential by 14.4% (Li et al. 2023).

Although cover crops and reduced tillage may imply a reduction in operational costs related to energy, they might induce a reduction in crop yield in the short and medium term (Glenk et al. 2017). On the other hand, cover crops maintain soil fertility in the longer term (Sun et al. 2011), thus signifying the need for policy intervention including financial incentives for farmers during the early stages of the transition from ploughing to conservation agriculture to offset potential yield reductions (Alskaf 2018).

Management practices including cover cropping and reduced tillage, as mentioned above, align with conservation agriculture through the improvement of soil and water quality by reducing runoff and leaching, enhancing water retention, and preventing soil erosion (Alskaf 2018; Warner et al. 2017). Accordingly, these measures should be targeted to geographic areas with higher erosion risk (e.g., hilly terrain and certain soil types) and where arable farming is found to contribute significantly to diffuse water pollution (Glenk et al. 2017). Weed management and pests including slugs were, however, identified as considerable challenges for reduced tillage adopters (Alskaf 2018). In this context, ley integration in arable rotation systems offset 27% of British agricultural emissions through SOC sequestration (Jordon et al. 2022), while simultaneously being adopted as a tool to control weeds which evolved to gain herbicide resistance (e.g., blackgrass; Jordon et al. 2022). The mitigation potential is lower at the European level when including leys in rotations with annual crops (i.e., 4 to 10%), according to Englund et al. (2023).

Soil amendment under specific edaphoclimatic conditions is considered to be a CO_2_ removal technique. Particularly, soil amendment in the form of spreading crushed silicate rocks such as basalt to croplands, known as enhanced rock weathering, has been shown to be an effective mitigation measure in acidic loamy soils (Kelland et al. 2020). This mitigation measure aims to accelerate natural geological processes of SOC sequestration (as it enhances SOC stocks by a factor of four) and reduces energy demands for milling (occasionally carried out on-farm; McAuliffe et al. 2017) and associated carbon emissions from the use of fossil fuels (Lefebvre et al. 2019). According to Kelland et al. (2020), this intervention is beneficial for both farmers and the environment since economic gains derived from yield improvement could offset the purchase and operational costs of enhanced rock weathering. Moreover, a supplemental source of silicon (Si), calcium (Ca), and potassium (K) can be provided without any increase in toxic trace elements. These elements, apart from improving crop production, increase protection from pests and diseases, and restore soil fertility and structure (Beerling et al. 2018). As a consequence, the well-managed soil amendment addresses multiple UN Sustainable Development Goals (Smith et al. 2019) and contributes to net-zero objectives.

Similarly, another important soil amendment is the replacement of ammonium sulfate with a different form of sulfur (S) (e.g., single superphosphate, potassium sulfate, magnesium sulfate, calcium sulfate dihydrate (gypsum), and polyhalite (polysulphate)), which are most notably observed on high pH soils (Powlson and Dawson 2022). With each of these S fertilizers, the content of phosphorus (P), K, magnesium (Mg), or Ca needs to be considered when deciding on other nutrient applications (Powlson and Dawson 2022). Elemental S can also be used, but it is more slowly available to crops than the other forms as it must first be oxidized to sulfate by soil bacteria and the rate of conversion is somewhat unpredictable (Malhi et al. 2005). This relatively easy measure would make a significant contribution to reducing NH_3_ emissions (i.e., by 90%; Powlson and Dawson 2022). Biochar application to soils has also been recommended as an important component of the pathway to “climate-smart soil” management practices in modern agriculture (Purakayastha et al. 2019). It has been shown to improve soil quality (soil bulk density, porosity, water retention, soil aggregation, and hydraulic conductivity; Purakayastha et al. 2019). Moreover, the increase in soil pH with biochar addition would result in a greater availability of primary and secondary nutrients like K, P, Ca, and Mg, as reported by Purakayastha et al. (2019).

Regardless of the pedoclimatic conditions, biosolid application to croplands provided valuable evidence in support of maintaining a sustainable agricultural landbank for biosolid recycling in the UK (Water UK 2010). Indeed, the mitigation measure helped to reduce up to 17% of GHG emissions (through SOC sequestration) in established experimental platforms at four sites in England with contrasting soil types and agroclimatic conditions (Nicholson et al. 2018). The mitigation potential through SOC accumulation in the latter study is comparable to 19% in Canadian croplands after biochar application (Gross et al. 2022). Moreover, biosolids amongst other environmentally positive impacts related to increasing water infiltration rate may improve soil quality and fertility. Biosolids contain valuable quantities of crop-available N, which can replace some of the required mineral fertilizer N together with increasing soil extractable P and total S for the plants (Rigby et al. 2016).

Acidification of digestate has been shown to be an effective mitigation measure for the utilization of food waste because it contributes to the mitigation of N losses (with around 95% reduction of cumulative NH_3_ losses, which indirectly produce N_2_O through microbial nitrification) following application to croplands (Sánchez-Rodríguez et al. 2018). This mitigation measure provides an environmentally sound option for N management and higher yields, as well as the production of renewable energy via anaerobic digestion (Kataki et al. 2017).

It is worth noting the importance of appropriate crop nitrogen management to avoid unnecessary trade-offs (e.g., potential increase in ammonia volatilization and nitrate leaching and ensure optimal crop production). In this context, Cammarano et al. (2021), for example, established an optimal N fertilizer rate of 120–140 kg N ha^−1^, in malting barley production in order to maximize the economic return, maintain acceptable grain N%, and minimize environmental impacts including marine and terrestrial eutrophication.

### Livestock systems

#### Manure management

Introducing anaerobic digestion to grassland-based livestock systems has demonstrated mitigation of the C footprint of livestock production (Webb 2017). For instance, the anaerobic treatment of dairy processing effluents showed a mitigation potential of 15.1 kg CO_2_-eq according to Stanchev et al. (2020). Likewise, via predictive modelling based on the IPCC refined methodology, Scott and Blanchard (2021) simulated up to 44% reduction of total commercial dairy farm emissions through the adoption of anaerobic digestion. This is in line with Battini et al. (2014), as anaerobic digestion can lead to an over 30% reduction in GHG emissions, compared to traditional manure treatment. Although its implementation can be challenging, especially for small farms (due to the cost) or those located with insufficient access to water (Smith et al. 2021), anaerobic digestion provides diverse positive environmental impacts. For instance, high bioavailable N from digestate enables lower inorganic fertilizer requirements per hectare (Walsh et al. 2018). In addition, the application of bio-slurry as an organic fertilizer increases SOC sequestration (Walsh et al. 2018). Moreover, it enables pollution control by removing waste from the environment and reducing N and P discharge to the waterbodies (Scott and Blanchard 2021), and reduces land occupation and ozone depletion (Stanchev et al. 2020).

As briefly mentioned above, anaerobic digestion can be expensive and requires improvements in the maintenance of digesters to avoid increased emissions (Smith et al. 2021). However, energy savings from anaerobic digestion are important (NFU 2021b). Such savings are estimated to reduce 715 t CO_2_-eq year^−1^ (41%) for commercial dairy farms in Northern Ireland (Scott and Blanchard 2021). Exploiting the CO_2_ component of biogas and the ability to use CH_4_ to power farm vehicles are seen as routes to achieve a reduction of 50% GHG emissions via offsetting (Scott and Blanchard 2021). Accordingly, government support could be instrumental in overcoming the costs of investment either using capital grants targeting the pollution reduction potential of systems or tax breaks and profitable tariffs to encourage the uptake of anaerobic digestion, thus providing renewable energy to the national grid (Scott and Blanchard 2021).

Applying additives to slurry (e.g., acidifiers alum, calcium chloride, and sulfuric acid) has shown abatements of NH_3_ emissions up to 76% from confined dairy production (McIlroy et al. 2019). However, the technologies for the application of these additives in livestock housing need to be further developed (McIlroy et al. 2019). It is important to note that abatement techniques for manure management involve a holistic approach and should be implemented at both the storage and land spreading stages (Montes et al. 2013).

#### Grassland management: fertilization and extensification

Several mitigation measures related to N fertilization have proved to be efficient in terms of GHG mitigation. For instance, organic amendment scheduling compared to a traditional one-time application per season may be a useful on-farm mitigation measure for minimizing N_2_O emissions (Shah et al. 2020). The use of high-frequency, low-dose organic fertilizer applications was predicted to reduce N_2_O peak fluxes (up to 17%) for cattle slurry during the autumn and spring seasons (Shah et al. 2020). Furthermore, the optimal use of organic fertilizers has potential benefits compared to synthetic fertilizers, as it enhances forage yield and livestock productivity and soil quality (through SOC storage) and provides high-value organic food production with a suitable source of bioavailable soil nutrient replenishment (Zheng et al. 2010; Wang 2014; FAO 2017).

The application of nitrification inhibitors during fertilization has been shown to mitigate soil emissions (Chadwick et al. 2018). For instance, dicyandiamide (DCD) reduced N_2_O emissions by ~13% under trampled grasslands and 53% under tractor compaction (Hargreaves et al. 2021). The reduction in N_2_O emissions is accompanied by a decrease in NO_3_ leaching and runoff, and NH_3_ volatilization, all of which are indirect sources of N_2_O (Cardenas et al. 2022). However, caution should be taken as issues have been raised when using nitrification inhibitors, as traces of DCD were found in milk when DCD was directly fed to animals (Welten et al. 2014). Further, swards from grasslands which received DCD have been reported to contain traces of DCD (Pal et al. 2016). Despite this concern, there is no defined threshold concentration for DCD in human-edible produce related to food safety as the compound has been reported to be non-toxic under typical application rates (OECD 2004).

Similarly, sodium chlorate (NaClO_3_) amendment showed substantial mitigation potential with more than 60% reduction in the net nitrification rate under agricultural soils (Fu et al. 2018). Likewise, inhibited urea with *N*-(*n*-butyl)thiophosphoric triamide (NBPT) was shown to decrease NH_3_ emissions within a range of 48–65% under grasslands in England and Wales (Carswell et al. 2019a). However, with no apparent yield differences compared to other N fertilizer sources (e.g., ammonium nitrate and urea), there is no economic incentive for the farmer to use the more environmentally acceptable option, unless externality costs are incorporated into fertilizer prices at the point of sale (Carswell et al. 2019a).

N fertilizer should be applied optimally through soil testing prior to applications when increasing yield potential. Perhaps, the most promising outcome of reduced N fertilizer input is the reduction associated with N leaching into waterbodies (which subsequently produces *indirect* N_2_O) and *direct* GHG emissions during manufacture, transport, and application (Harris and Ratnieks 2022). The substitution of fertilizer nitrogen with symbiotically fixed nitrogen from legumes (e.g., white clover, *Trifolium repens*) within the range of 30–50% enables mitigation up to 58% g N_2_O‐N kg^−1^ DM yield compared to a baseline with a high fertilizer rate of 200 kg N ha^−1^ year^−1^ (Fuchs et al. 2020). This specific mitigation measure seems beneficial with respect to multiple outputs such as yields, N yields, and feeding values (Lüscher et al. 2014; McAuliffe et al. 2018), thereby improving animal health and welfare, through enhanced nutritional benefits (Carswell et al. 2019b). Indeed, root-node fixed N provides a supply of N for plants that is more bioavailable than occasional fertilizer applications and increases N use efficiency (Barneze et al. 2022) while improving diet-level sustainability (Costa et al. 2021). The biologically fixed N reduces energy costs associated with producing synthetic fertilizer with no reduction in productivity (Harris and Ratnieks 2022). Moreover, introducing local legumes has shown feasibility for replacing imported soy-based feeds, as reported by Costa et al. (2021). However, a potential limitation of this mitigation measure can be the challenge of achieving high and persistent legume proportions, particularly under grasslands receiving low sunlight or excessively cold growth periods (Barneze et al. 2022).

Moving towards extensification by reducing the livestock density and N fertilization has been underscored as a reliable mitigation measure (Sándor et al. 2018). The latter demonstrated a reduction of 78% in soil N_2_O emissions for the mown and grazed site of Easter Bush (Edinburgh; Sándor et al. 2018). The reduction in soil N_2_O emissions is within average estimations (~70%) for grasslands under similar conditions (e.g., France and Switzerland) (Sándor et al. 2018). The mitigation was accompanied by positive implications such as decreases in NH_3_ losses and NO_3_ leaching, thereby simultaneously reducing indirect N_2_O emissions (Sándor et al. 2018). On the other hand, intensification, and the specialization in livestock production, for example, dairy systems, results in both an increase in C footprint, which relies on feed importation, and burdens such as eutrophication and acidification (Soteriades et al. 2019). The effect of ongoing trends in dairy farms can be mitigated by (i) increasing beef output per unit of milk achievable without a large change in a dairy farm’s management and (ii) sustainable intensification of displaced beef-breeds production on suckler-beef farms (Soteriades et al. 2019). These measures can spare larger areas of land for forest (regionally or in major beef-exporting countries such as Brazil; Styles et al. 2018). Although this may reduce by up to 11–56% of burdens (i.e., GWP, eutrophication potential, acidification potential, and land occupation) per liter of milk (Soteriades et al. 2019), the investment in technology to maintain production levels and improve environmental efficiencies can be financially restrictive due to initial capital investment requirements (Dumont et al. 2013). Moreover, the positive environmental impacts of lower eutrophication and acidification potential could be negated by an increase in indirect land occupation related to animal feed cultivation (Gonzalez-Mejia et al. 2018).

Finally, regarding pork production, partly outdoor organic production where pigs spend part of the year outside and the rest indoors (seasonal housing) showed lower acidification, and thereby fewer indirect GHG emissions, than indoor systems. Conversely, traditional or “hardy” pig breeds which spend their lives outdoors yearly produce higher eutrophication potentials than semi-outdoor systems (Rudolph et al. 2018).

### Livestock diets

#### Supplements to inhibit greenhouse gas production

While the use of biotechnological interventions can be challenging on a practical basis, feed additive supplementation appears to be the most researched and therefore the most “ready-to-use” mitigation measure to mitigate enteric CH_4_ emissions and/or N_2_O emissions for ruminants (Prathap et al. 2021). For instance, dietary nitrate and increased lipids included together could reduce enteric CH_4_ emissions by 45% for finishing beef cattle (Duthie et al. 2018). This measure is achievable through the utilization of by-product feed such as rapeseed cake (Duthie et al. 2018). Potential adverse effects such as toxicity and impaired animal performance can be avoided by feeding low amounts of nitrate (Lee and Beauchemin. 2014). On the contrary, feeding nitrate to animals may increase N in excreta and therefore the trade-off between CH_4_ and N_2_O emissions reductions requires further research (Beauchemin et al. 2020). Similarly, supplementing dairy cow diets with oilseed-based preparations (e.g., extruded linseed or calcium salts of palm or linseed oil) as 22 g oil kg^−1^ DM showed a reduction of 10% of CH_4_ emissions per kilogram of DM (Kliem et al. 2019). In a meta-analysis, Arndt et al. (2022) showed that feeding oils or fats versus oilseeds had comparable mitigation effects on total daily CH_4_ production, with an average of 21% (ranging from 12 to 35%). This specific oilseed-based dietary mitigation measure is commercially practical with no negative effect on DM intake or milk fatty acid concentration (Kliem et al. 2019). However, it should be noted that feeding higher levels of oil supplements (≥50 g oil kg^−1^ DM) can have a negative impact on ruminal and total tract organic matter and therefore neutral detergent fiber (NDF) digestion (Firkins and Eastridge 1994). Furthermore, using 2 g of liquorice extract for feeding animals (rich in prenylated isoflavonoids and particularly glabridin) might potentially improve the efficiency of N utilization and reduce CH_4_ production in the rumen (Ramos-Morales et al. 2018). In this context, Ramos-Morales et al. (2018) conducted experiments which showed a reduction of 77% NH_3_ emissions and 27% CH_4_ emissions following the inclusion of 2 g extract of liquorice for sheep diets. The mitigation effect was accompanied with an improvement in feed conversion efficiencies by ruminants which subsequently increased their productivity (e.g., kg average daily gains). The invention of feed composition for ruminants comprising bis esters of hederagenin or ivy sapogenins (saponins are naturally occurring compounds that are widely distributed in all cells of legume plants) helped to mitigate ruminant emissions (Al Dulayymi et al. 2017). The synthetic molecule derives its name from its ability to form stable, soap-like foams in aqueous solutions and constitutes a complex and chemically diverse group of compounds including glycoside. The hederagenin bis esters have a persistent effect against ciliate protozoa in the rumen, without affecting the bacterial microflora, and feeding ruminants with doses of 50 mg to 1 g per kg per feed demonstrates a mitigation potential of up to 23% for enteric CH_4_ emissions and up to 16% for NH_3_ emissions (Al Dulayymi et al 2017). This dietary mitigation measure also helped to improve milk production and ruminant growth performance was observed to be more efficient (Al Dulayymi et al. 2017). In this context, several studies with saponins reported reduced CH_4_ production from ~6 to 27% by reducing the protozoa population (Goel and Makkar 2012).

Other effective supplements for reducing enteric CH_4_ emissions include concentrate supplementation with ground corn, essential oils, or acidic supplements (e.g., *encapsulated fumaric acid*) as well as certain plant secondary metabolites (e.g., grape marc; Prathap et al. 2021). Notably, a potential CH_4_ inhibitor known as 3-nitrooxypropanol (3-NOP) is receiving much attention. 3-NOP has been shown to be effective in long-term studies with dairy and beef cattle (Melgar et al. 2020). 3-NOP decreases CH_4_ production by 30% (Dijkstra et al. 2018; Kebreab et al. 2023). In general, the reduction of CH_4_ emissions derived from enteric fermentation is within the large range of mitigation reported by UNEP (2021) at a global scale (i.e., 15 to 45%). However, farmers should be selective regarding this feeding practice, as some of the feed additives might be expensive (e.g., propionate precursors) or have side effects such as reduced calorie intake (e.g., halogenated compounds; Smith et al. 2021).

#### Modifying feeding regimes

Replacing a moderate proportion of total mixed ration-based diets with freshly cut and delivered grass or grass grazed at pasture for dairy cows showed a reduction in CH_4_ emissions of up to 17% for the animals fed fresh cut grass and up to 39% for the grazing animals (Cameron et al. 2018). Within this mitigation measure, the costs of any longer-term reductions in milk yields may be outweighed by the benefits of improved farm profitability and reduced GHG emissions (Cameron et al. 2018). High-sugar grasses are thought to provide a better balance of N and carbohydrates to rumen microbes, thereby improving N and feed efficiency (Soteriades et al. 2018). In this context, re-seeding conventional permanent pastures (which occupy ~70% of UK-based agricultural land) with high-sugar grass varieties is seen as an attractive short-term measure for farmers by improving productivity, and reducing acidification and eutrophication impacts. However, it is important to note that primary data (e.g., digestibility and crude protein measurements as well as animal growth rates during grazing)–based assessments of high-sugar grass introduction suggest that the cultivar may produce more N_2_O emissions and poorer animal performance compared to other swards such as those including legumes (e.g., white clover; *Trifolium repens*) under clayey soil types and temperate climatic conditions (McAuliffe et al. 2018; Mcauliffe et al. 2020b).

Grazing of dairy cows has also been shown to be effective with respect to SOC sequestration (Wilkinson et al. 2021). Pasture access benefits milk quality (i.e., milk produced on grass has higher levels of digestible protein as well as vitamin E and carotene; Wilkinson et al. 2021). Grazing dairy cows display behaviors including improved lying/resting times, lower levels of aggression, more normal estrous behaviors, and better synchronicity of behaviors compared to housed cows (Mee and Boyle 2020). Farmers are thereby encouraged to provide pasture access to dairy cows whenever weather conditions permit. Nevertheless, ruminant welfare is complex and there are different schools of thought about benefits and risks related to year-round housing, but when managed appropriately, improved welfare through grassland access has been shown to improve productivity and therefore reduce GHG emissions via fewer CH_4_ and N_2_O emissions (Rivero and Lee 2022). It is also worth mentioning that improving welfare (e.g., reducing lameness occurrences, preventing liver fluke, reducing stocking densities, and minimizing tuberculosis outbreaks) can actually marginally increase GHG emissions in certain livestock systems such as poultry while reducing water and soil pollution (Leinonen et al. 2014). In the case of ruminants, unintended consequences of improved animal welfare include reduced gross margins due to increased management costs (Rivero and Lee 2022). These complexities require further investigation to determine (a) whether the observed trade-offs can be balanced through mitigation measures and management practices (e.g., cell-grazing for ruminants) or (b) if one aspect of sustainability (i.e., environmental benefits, animal welfare improvements, or increased profitability) should be prioritized over the others. To add to the aforementioned complexities, other studies have demonstrated that improved profitability via high-quality management practices (e.g., high levels of feed conversion ratios) can in fact improve environmental health and economic performance simultaneously in intensive pig production systems (McAuliffe et al. 2017); despite this encouraging finding, implications for animal welfare require further exploration in the context of achieving net zero (see Section 3.4 for more information).

In terms of point (a) in the previous paragraph, feeding Ericaceous species (e.g., plants which thrive in low pH soils) to grazing sheep and red deer on heathlands is an effective mitigation measure to mitigate GHG emissions (Pérez-Barbería et al. 2020). Indeed, the mitigation measure balanced multiple trade-offs through improved cost-effectiveness, reduced the C footprint, and demonstrated biodiversity gains compared to other systems of animal production such as intensive farming (when animals are indoors, and fed on imported food and silage) (Gordon and Prins 2008). Ericaceous species also help to maintain traditional grazing culture and improve animal welfare (Pérez-Barbería et al. 2020). On the other hand, introducing high concentrate (e.g., barley or maize based) diets fed to different breeds of beef cattle during the finishing period helped to reduce up to 45% of CH_4_ emissions, while increasing feed efficiency and propionate (a main precursor of glucose for ruminants) production, thereby decreasing CH_4_ production in the rumen (Snelling et al. 2019).

Nevertheless, under grazing systems, larger areas of pasture may be needed to produce the same amount of throughput (Wilkinson et al. 2021). In this context, diets for livestock could be formulated to reduce the total feed-related C footprint and reduce the proportion of human-edible feed in the total diet (Wilkinson and Garnsworthy 2017). For instance, dairy cow diets formulated to include high proportions of by-product feeds such as dried distillers’ grains can support high levels of milk output and are environmentally attractive compared with those based on grazed pasture or silage with concentrates (Wilkinson and Garnsworthy 2017). By-product utilization contributes to a circular economy via waste avoidance and reduction of “empty” (i.e., agricultural produce which ends up in landfills, incinerators, or slightly less burdensome, recycling centers) GHG emissions.

### Livestock health and genetic performance

Although highly complex in terms of sustainability trade-offs, as introduced in Section 3.3, improving livestock health has been shown to have positive environmental and societal benefits in certain livestock systems; for instance, the reduction of GHG emissions arising from livestock production can be delivered by reducing the maintenance of poorly performing animals through genetic selection (Llonch et al. 2017; McAuliffe et al. 2018). Improving health can lead to improvements in the parameters that ruminants’ emission intensities are sensitive to, e.g., maternal fertility, abortion rates, and cow mortality rates, while calf, ewe, and lamb mortality rates and growth rates, milk yields, and feed conversion rates are also important factors to improve (MacLeod and Moran 2017). Regarding C “credits,” the marginal cost for livestock health improvement was higher than −100 £ t^−1^ CO_2_-eq for cattle and lower than 50 £ t^−1^ CO_2_-eq for sheep production (MacLeod and Moran 2017). Similarly, performance recording technology showed livestock production’s potential to be C efficient, thus adhering to growing public demands on climate change and animal welfare simultaneously (Morgan-Davies et al. 2021). For instance, using performance recording on sheep farms in order to achieve higher genetic merit mitigated up to 18% of GHG emissions (3.5 CO_2_-eq kg liveweight^−1^) and increased economic margins by £6 ewe^−1^, thereby ensuring enhanced food security and lower climate-related impacts; however, this management practice incurred 10% extra labor with ramifications for profit-loss margins (Morgan-Davies et al. 2021). Moreover, future animal breeding schemes may include a wider range of traits linked to environmental emissions apart from production and health traits (Gill et al. 2021). Wallace et al. (2019) reported that a heritable subset of the core rumen microbiome dictates dairy cow productivity and CH_4_ emissions. As alluded to above, in theory, it should then be possible to select ruminants with specific rumen microbiomes suited to different production systems, leading to higher feed efficiency (e.g., through increased digestible energy) and lower CH_4_ emissions. This is a notable finding as improvements to the biological performance of ruminants fall behind the performance of monogastrics which are easier to increase feed conversion efficiencies due to the absence of rumen microbial communities.

Considering livestock bedding material, straw is commonly used and often transported long distances from arable to livestock regions (Copeland and Turley 2008). This process is becoming increasingly unsustainable and uneconomical as the demand and price for straw increase (Wonfor 2017). Alternative bedding materials (for instance, coppice willow and miscanthus) cultivated directly on livestock farms could potentially avoid transport-related emissions and competition for use (Glithero et al. 2013). In this context, the use of miscanthus bedding production on livestock farms and the substitution of fossil fuels with straw in electricity generation have been shown to provide environmental benefits (Yesufu et al. 2020). This mitigation measure is considered to be cost-effective and capable of reducing GHG emissions by ~9 million t CO_2_-eq at a UK level and also minimizes both eutrophication and acidification burdens (Yesufu et al. 2020).

### Horticultural systems on peatlands

Around 40% of UK peatlands have been drained for agricultural use, namely horticultural cultivation, which has caused serious peat wastage and associated GHG emissions (CO_2_ and CH_4_; Dixon et al. 2014). While peatland drainage increases CO_2_ loss into the atmosphere, natural peatlands are sources of CH_4_ due to methanogenic activity under their prevalent waterlogged anoxic soil conditions. To address GHG emissions and C losses, water tables should be raised (or lowered if applicable) to reduce GHG emissions from agricultural peatlands while simultaneously maintaining the current levels of horticultural productivity (Musarika et al. 2017). For instance, increasing the water table to −40 cm presented a possible compromise to decrease peat oxidation and maintain romaine lettuce production (Matysek et al. 2022). Similarly, raising the water table from −50 to −30 cm in lowland fen peatland used for radish production reduced GHG emissions (i.e., CO_2_ by 89% and CH_4_ by 58%), while maintaining the same yield production (Musarika et al. 2017). Likewise, maintaining a high-water table in different horticultural peatlands helped to reduce the global warming potential by approximately 30% (Taft et al. 2018). However, it is important to bear in mind that this mitigation measure may be impractical to implement within current horticultural systems. For instance, raising the water table to within 15 cm of the soil surface would not be implemented while a crop was in place, as it would likely result in high crop mortality and thus be unsuitable for field trafficking. Instead, this intervention would probably need to be implemented between summer crops, possibly over quite short fallow periods (Taft et al. 2018). Optimizing the water table in agricultural peatlands contributes significantly to economic development in many areas (Evans et al. 2021) and promotes food security (Taft et al. 2018).

### Mixed farm systems and their role in sustainable agriculture

Integrated farming under horticultural and crop systems has demonstrated the capability to mitigate more than 100% of GHG emissions, while enhancing food health and promoting agricultural sustainability (Abdul-Salam et al. 2019). Integrated farming involves cover crops, legumes, conservation tillage, reduced mineral fertilizer, pesticide and herbicide applications, and soil amendments to increase SOC content. However, since the relative financial performance of conventional farm systems is better than many low-carbon integrated farm systems, price premiums of up to 20% for integrated farming would help to enhance their economic performance to be comparable with conventional farming (FWI 2017; Abdul-Salam et al. 2019). In this way, consumers are increasingly sourcing low-carbon produce and paying extra as a way of improving their food health and contributing to reductions in their C footprints (Abdul-Salam et al. 2019).

Under both croplands and grasslands, several practices could be implemented to maximize crop nutrient utilization and to minimize emissions to the environment. As an “environmentally benign” material, applying green/food composts (characterized by lower N content, compared to food digestate and slurry) reduced N_2_O emissions by up to 54% while accumulating long-term soil organic N reserves and improving soil structure and nutrient composition (Nicholson et al. 2017). Farmers are also advised to apply food-based digestate, as a provider of renewable energy, in the spring where practically possible, or in autumn to an actively growing crop such as grass or oilseed rape (Nicholson et al. 2017). Under this management, the crop will take up available N from the soil which will not be lost via overwinter NO_3_ leaching (Nicholson et al. 2017). Similarly, bandspreading is thought to be effective at reducing NH_3_ emissions (up to ~70%) from slurry instead of surface broadcasting (Nicholson et al. 2017). Precision application (i.e., bandspreading) provides numerous other advantages over broadcast applications: for example, more accurate assessment of application rates, the ability to apply from tramlines, reduced odor and crop damage, and a cleaner sward can be achieved (Nicholson et al. 2017). However, the effectiveness of this technique is dependent on the prevailing soil conditions (Nicholson et al. 2017).

Within arable and livestock systems, when using the by-products of whisky production to replace alternative feed ingredients (such as imported soya meal) for livestock, notable reductions of GHG emissions were shown (associated with land use changes, and to a lesser extent with enteric fermentation, manure management, and the end use of manure and its potential to replace synthetic fertilizers) (Leinonen et al. 2018). As briefly discussed in Section 3.3, distillery by-products could also be used as anaerobic digester feedstock to generate renewable energy (heat and electricity), though the mitigation potential as animal feed is lower than using it as human-edible ingredients (0.703 to 0.759 kg CO_2_-eq kg^−1^ DM of by-product used for human consumption, compared to 0.101 to 1.219 kg CO_2_-eq kg^−1^ DM of by-product used for animal feed; Leinonen et al. 2018). When used as an organic fertilizer, digestate arising from the anaerobic digestion process is high in N and P, as well as C, thereby simultaneously accumulating SOC *and* reducing the need for synthetic fertilizers (Leinonen et al. 2018), which are a major source of agri-food related GHG emissions.

### Offsetting greenhouse gas emissions on agricultural land

Agroforestry systems deliver environmental benefits through C uptake compared with grasslands or croplands without trees (Jordon et al. 2020). Agroforestry, including silvopasture systems, shelterbelts, windbreaks, riparian buffer strips, hedges, wood pasture, forest grazing, orchards, woody biofuel, and farm woodlands, is gaining considerable attention from the perspective of agricultural sustainability, particularly in terms of net-zero ambitions globally. For instance, in terms of GHG mitigation and SOC sequestration, forest regeneration on sheep pasture with natural regeneration or forest plantation showed a mitigation potential of up to 85 t CO_2_-eq ha^−1^ and 147 t CO_2_-eq ha^−1^, respectively, over 25 years (O’Neill et al. 2020). Moreover, planting red alder trees into sheep-grazed pasture showed a CO_2_ mitigation potential of 47.5 to 99 Mg C ha^−1^, after 20 years, for different types of red alder trees (Nworji 2017). Likewise, land use change by either afforestation with species of broadleaf trees (planted at 800 or 1600 stems ha^−1^), or reversion to rough grassland, showed both soil N and C accumulation increasing SOC up to 46% and 334%, respectively, for 21 years (Baddeley et al. 2017). When pragmatically feasible, establishing hedgerows and field margins in arable landscapes and agroforestry systems could provide up to 63 t C ha^−1^ (Dunn et al. 2021). The mitigation potential is comparable to the estimated 81.7 ± 28.8 t C ha^−1^ for hedgerows in Belgium (Van Den Berge et al. 2021). Similarly, Crous-Duran et al. (2020) using modelling showed that introducing trees in arable systems allowed the sequestration of up to ~400 t C ha^−1^ in high tree-density agroforestry systems. Likewise, Poulton et al. (2018) analyzed rates of SOC increase in the treatments on 16 long-term experiments in the southeast UK. The latter study showed that the conversion from cropland to grassland or woodland enhanced SOC sequestration exceeding *4 per 1000* SOC stocks per year in the case of woodlands and reaching 55% in the case of grasslands. More widely, under the European territory, agroforestry implementation in the priority areas (areas with the highest number of accumulated pressure), which made up 8.9% of total European farmland, would reduce between 1.4 and 43% of European agricultural GHG emissions, depending on the type of the agroforestry (Kay et al. 2019). In addition, several environmental impacts could be reduced under agroforestry systems due to microclimate amelioration through the windbreak effect of the trees, the conservation of soil and water, and wildlife habitats as well as the forest productivity and sustainability through C uptake, thereby GHG offsetting contributing to cross-sector net-zero targets (Nworji 2017; Jordon et al. 2020).

It is worth noting that the viability of land use conversion to agroforestry, without subsidies, depends on low farm performance, a strong likelihood of natural regeneration, and a high carbon-market price. For instance, Burgess and Rosati (2018) confirmed that silvopastoral systems are not financially profitable (compared to silvoarable systems) but they provide the greatest societal benefit if environmental externalities are included. Accordingly, imposing, e.g., carbon payments or penalties for nutrient or soil loss pollution, would make agroforestry a more financially profitable opportunity for sustainable food production and security (Kay et al. 2019). In other words, financial aid for woodland establishment, a strategy being deployed in the UK by the “Woodland Trust,” makes planting trees to sequester C financially viable (O’Neill et al 2020). However, other studies, such as Crous-Duran et al. (2020), showed that introducing trees in different farming systems such as arable and pasture, as a solution for additional environmental benefits, maintained similar levels of productivity. Afforestation mitigation measures provide economic benefits in terms of monetary value (e.g., harvesting wood for paper pulp or heating fuel which would offset fossil fuel depletion and associated GHG emissions), job creation, and financial income for rural economies as well as contributing to the circular economy if managed appropriately (Dunn et al. 2021). Many of the “tree outputs” have different established markets such as timber, food, energy, recreation, and non-timber forest products (e.g., foliage, biochar, and Christmas trees), which offer a developing or niche opportunity for farm enterprises to enhance ecosystem services (Pagella and Whistance 2019). Decision support tools should be offered at the planning stage of farm woodland schemes to aid farmers in tree species selection and assessment of benefits and trade-offs (Wiik et al. 2019). It is also important to bear in mind that the rate of SOC increase slows as the new equilibrium value (i.e., reaching SOC saturation) is approached and that increases are reversed if the modified management practices are not continued (Smith 2014).

Widespread adoption, however, would have a negative impact on global food security, e.g., converting agricultural land to forest or grassland (Poulton et al. 2018). Conversion to grasslands and woodlands could be convenient in limited situations where soils are either of low productivity or are fragile and prone to erosion, to ensure food security (Albanito et al. 2016). Moreover, afforestation should be accompanied by a shift in diet away from meat and dairy products. This change is necessary because without it, it would be necessary to import additional meat and dairy products from overseas (Dunn et al. 2021).

### Bioenergy production

The CCC identified that bioenergy coupled with carbon capture and storage (BECCS) could deliver a significant reduction of up to 53 Mt CO_2_-eq by 2050 (BEIS 2021). Indeed, bioenergy crops help mitigate climate change through displacing fossil fuel energy generation while removing CO_2_ from the atmosphere and storing it in soils. This is the case with willow and miscanthus which both offer biomass production and higher SOC sequestration rates (with up to 12% increase in soil depths of 0–0.3 m) when planted in arable soils (Gregory et al. 2018). Robertson et al. (2017) estimated that the miscanthus-derived soil C accumulated a rate of 860 kg C ha^−1^ year^−1^ over the top 30 cm. Therefore, miscanthus cropping could be attributed as a CO_2_-sink related to an additional credit from soil C sequestration in the soil during the cultivation period, as confirmed in the Felten et al. (2013) study in Western Germany. Harris et al. (2017) showed that the conversion of grassland to short rotation coppice bioenergy willow converted the system from a net C source of 119 g C m^−2^ year^−1^ to a net sink, −620 g C m^−2^ year^−1^.

However, in the UK, conversion of grassland to bioenergy cropping systems represents one of the most significant potential land use transitions, as grasslands are a considerable part of the UK landscape (4–5 10^6^ ha; Defra et al. 2007) and management of grasslands can vary widely in the UK, particularly with respect to fertilizer input and grazing strategies (Harris et al. 2017). As a consequence, it is desirable that bioenergy crops are concentrated on less-productive “marginal” land to minimize conflict between food and bioenergy production on higher-quality soils (McCalmont et al. 2017).

Lastly, poultry litter has been shown to perform better than miscanthus for most of the impacts. In this sense, gasification of poultry litter to produce electricity and heat generation in the UK could save 1.7 Mt CO_2_-eq year^−1^, equivalent to around 0.4% of UK’s GHG emissions (Jeswani et al. 2019). However, owing to high capital costs, the unsubsidized cost of generating heat and electricity from poultry litter is similar to that of natural gas heat and power but significantly cheaper than that from other fossil fuel alternatives within an abatement cost of £34 t^−1^ CO_2_-eq. This signifies that animal waste (by-product) management is a critical research stream in the context of agriculture’s contribution to a net-zero economy.

## Limitations and critical gaps for future research

### Limitations

Our findings on GHG mitigation measures applied in the UK are applicable to broader geographies under similar climatic conditions. Despite adhering to a standard operating procedure for systematic reviews, our synthesis of results did not apply streamlined effect size predictions of the benefits and risks surrounding individual (or combined) GHG mitigation measures as the data extracted was not consistent in terms of agricultural systems, mitigation measures, and edaphoclimatic conditions in the UK (Jebari et al. 2023); as a result, this made statistical analyses of these reviewed measures’ potential to contribute to the UK’s net-zero ambitions infeasible. Likewise, emission reductions were provided per area or per kilogram of product. However, emission reductions per area may imply a caveat associated with reductions in productivity. Further, although the resulting dataset provides novel information to guide future research in the context of agriculture’s net-zero achievements, the results should be interpreted with caution as they could potentially be misleading within the study’s geographic boundary due to the low UK-specific literature sample size (*n* = 52). Despite this limitation, the resultant dataset (Jebari et al. 2023) provides a simple, yet comprehensive progress to communicate cutting-edge sustainability research with the farming community, thereby enabling qualitative analyses to guide future scientific efforts which are economically (e.g., capital investment requirements) and socially feasible.

### Critical gaps for future research

As touched upon throughout the examination of literature, knowledge gaps were highlighted in our findings related to the implications of various mitigation opportunities for the UK’s agricultural systems. While the environmental impacts of different mitigation measures have been investigated extensively, other impacts remain poorly understood. For instance, barriers on the adoption of the mitigation measures for the farmer, in terms of ease of maintenance or installation and operational costs, have been overlooked by 49% of the reviewed literature (see dataset; Jebari et al. (2023)). In this context, information on the attitudes of farmers towards the different management practices is needed (Collins et al. 2016), as farmers make the management decisions for most agricultural land in the UK (Harris and Ratnieks 2022). Engaging farmers on the issue of climate change mitigation (e.g., via participatory extension programs, surveys, and workshops, where farmers are allowed to share their feedback) is one option to address this current important knowledge gap (Knook et al. 2020). This bridge between scientists and farmers has already been established as part of another complementary, collaborative, and nationwide research stream which aims to identify which mitigation measures should be explored more rigorously from the agricultural community’s perspective (see Section 4.1).

Moreover, the energy implications of the mitigation measures (i.e., whether the mitigation measure implies energy consumption reductions or increases) were not considered in 52% of the studies reviewed herein, even though entire food supply chains are major energy users and contributors to climate change (Rosa et al. 2021). Similarly, food security provision was overlooked in 51% of the studies reviewed, despite the potential negative trade-offs between food security and climate mitigation (Fujimori et al. 2019). Particularly, the import requirement induced by the mitigation measure was stated in only 15% of the retained studies.

Although the financial viability (in monetary and/or productivity terms) of the mitigation measures was considered in most of the studies reviewed (> 77% of studies), the marginal abatement cost (i.e., the average cost of reducing 1 ton of CO_2_ equivalent) was rarely considered. The latter was not mentioned in 90.6% of the studies, which could be considered a major knowledge gap for future research. The cost-effectiveness of mitigation measures can change in response to factors such as commodity prices and the indirect effects of non-GHG policy (MacLeod et al. 2010). Even though prices and/or costs are fluctuating with time (Tang et al. 2021), marginal abatement cost information of potential mitigation measures has been shown to help policymakers identify the most recent cost-effective GHG mitigation options (Eory et al. 2018). As a consequence, the generation of accurate information on the cost-effectiveness of the mitigation measures is needed for effective government policies.

## Conclusions

We synthesized existing evidence for several agricultural management practices and technologies, which can be deployed on farms, in order to help mitigate climate change. In many cases, the mitigation measures provided co-benefits for farmers, including improving farm productivity and diversifying farm income through energy generation. Well-implemented measures also result in environmental co-benefits in addition to mitigating climate change, including biodiversity, soil health, and other ecosystem services related to human health and animal welfare. However, it is also important to look at the sustainability from the farmers’ perspective. Uneconomic practices for farmers (e.g., bioenergy industrial plants, agroforestry establishment) could be potentially overcome by government changes in regulations and subsidies to ensure greater financial viability by compensating for initial high capital costs. We have synthesized the evidence base within existing literature (Jebari et al. 2023), primarily focusing on the relevance to the UK’s GHG strategies up to 2050 and the identification of opportunities and risks which require further attention. Our open-access dataset (Jebari et al. 2023) can inform scientists and policymakers on state-of-the-art GHG-related studies and guide funding bodies to target areas, which need urgent attention. Finally, net-zero achievement and relevant government policies need to be examined more holistically (e.g., accounting for unintended consequences such as farmers’ well-being and animal welfare) in the context of business resilience and broad sustainability. This is particularly pertinent to food security as there is an ever-increasing population, which only the agri-food sector as a whole can sustain.

### Supplementary Information

Below is the link to the electronic supplementary material.

Supplementary file1 (DOCX 119 KB)

## Data Availability

The datasets generated and/or analyzed during the current study are available in the Mendeley repository, Jebari et al. (2023) Dataset on agricultural greenhouse gas mitigation measures in the UK, Mendeley Data, V1, https://doi.org/10.17632/t9kynfj5jf.1.
