# Supplementary material for: Feasibility of mitigation measures for agricultural greenhouse gas emissions in the UK. A systematic review
Source: Agron Sustain Dev. 2023 Dec 28;44(1):2. doi: 10.1007/s13593-023-00938-0 (PMC10754757; doi:10.1007/s13593-023-00938-0)
Supplement: Supplementary file 1 — Supplementary file1 (DOCX 119 KB) [file 13593_2023_938_MOESM1_ESM.docx]

**Feasibility of mitigation measures for agricultural greenhouse gas emissions in the UK: a systematic review**

**Asma Jebari^a*^, Fabiana Pereyra-Goday^b^, Atul Kumar****^a^,** **Adrian L. Collins^a^,** **M. Jordana Rivero^a^, Graham McAuliffe^a^**

*^a^Net Zero and Resilient Farming, Rothamsted Research, North Wyke, Okehampton, Devon, EX20 2SB, UK*

*^b^Instituo Nacional de Investigacion Agropecuaria (INIA), Ruta 8 km 281, Treinta y Tres (postcode 33000), Uruguay*

***Corresponding author:** Asma Jebari

**Email address:** asma.jebari@rothamsted.ac.uk

**Table 1** *Summary of the mitigation measures related to emission reduction and/or production efficiency*

| A: Grazing management | |
| --- | --- |
| Mitigation measure | Implications |
| 1: Extensification: Decreasing inputs (nitrogen fertilization and livestock density) | - **Value of GHG mitigation**: 40 to 65% reduction in GHG emissions of managed grasslands (Sándor et al., 2018) Up to 70.6% reduction in N_2_O-N emissions (Case of N reduction) (Sándor et al., 2018) - **Energy**: Decrease in energy use (Clora et al., 2021) - **Productivity and/ or cost**: Increase in net primary production per animal (Sándor et al., 2018) - **Abatement cost**: Not available in the covered literature - **Environmental impacts:** Ecosystem sustainability (Sándor et al., 2018)   Carbon and nitrogen sequestration (Sándor et al., 2018)  Decrease in NH_3_ pollution and nitrate leaching (Sándor et al., 2018) |
| 2: Sustainable intensification: Increasing dairy farm diversification via higher dairy-beef output and intensification of displaced beef production | - **Value of GHG mitigation:** Up to 56% in C footprint L^-1^ of milk (Soteriades et al., 2019) - **Energy:** Not available in the covered literature - **Productivity and/ or cost:** Little economic incentive for farms to increase their beef: milk ratio at current milk and beef prices (Soteriades et al., 2019) - **Abatement cost**: Not available in the covered literature - **Environmental impacts:** Minor increase in resource depletion potential Lower eutrophication potential, acidification potential (Soteriades et al., 2019)   Increase in indirect land occupation related to concentrate cultivation Air pollution via NH_3_ as a result of the intensification and manure storage (Soteriades et al., 2019)  Replacing extensive UK beef production, enabling afforestation on less productive grassland, or by avoiding expansion of Brazilian beef production (Styles et al., 2018)   - **Challenges or trade-offs:** Investment in technology can maintain production levels and improve environmental efficiencies in intensive systems, although such technologies can be capital-intensive (Soteriades et al., 2019) |
| B: Manure management | |
| 1: Anaerobic digestion | - **Value of GHG mitigation**: Up to 44% reduction in total GHG emissions (Scott and Blanchard, 2021) - **Energy**: Biogas production, as a renewable energy. Energy saving up to 41% (Scott and Blanchard, 2021; Leinonen et al., 2018) - **Productivity and/ or cost**: Costly (Scott and Blanchard, 2021). Little incentive for widespread adoption of manure-based farm-scale anaerobic digestion in the UK (Scott and Blanchard, 2021) - **Abatement cost**: Not available in the covered literature - **Environmental impacts:**  Higher available nitrogen from digestate enables lower inorganic fertilizer to use per hectare (Scott and Blanchard, 2021) Pollution control (Scott and Blanchard, 2021; Stanchev et al., 2020) - **Challenges or trade-offs:** Complexity for small farms (Scott and Blanchard, 2021)   Implementation of anaerobic digestion must be combined with improvements in maintenance of digesters in order to achieve maximum benefits in CH_4_ emission reduction and avoid increased emissions (Smith et al., 2021)  Anaerobic digestion can only be implemented in locations with sufficient access to water (Smith et al., 2021) |
| C: Livestock feeding regime and breeding practices | |
| 1: Feed additive supplementation (e.g., lipid and nitrate dietary, extract of liquorice^2^, oilseed-based preparations^3^) for ruminants | - **Value of GHG mitigation**: Up to 45% reduction in CH_4_ enteric emissions (e.g., case of lipid and nitrate dietary) (Duthie et al., 2018)   Up to 77% reduction in NH_3_ emissions (e.g., case of liquorice) (Ramos-Morales et al., 2018)   - **Energy**: Not available in the covered literature - **Productivity and/ or cost**: Improves livestock productivity (Kliem et al., 2019; Al Dulayymi et al 2017) - **Abatement cost**: Not available in the covered literature - **Environmental impacts:**  Improves the efficiency of the feed utilisation by ruminants (Ramos-Morales et al., 2018)   Reduces NH_3_ urine excretion (Al Dulayymi et al., 2017)   - **Challenges or trade-offs:** Feeding higher levels of supplements can have a negative impact on ruminal and total tract organic matter and NDF digestion (≥50 g oil/kg DM oilseed-based preparations) (Kliem et al., 2019)   Risk of toxicity and impaired animal performance by feeding higher amounts of dietary supplements (e.g., lipid and nitrate dietary) (Duthie et al., 2018) |
| 2: Modifying feeding regime  Ruminant: Increasing the use of fresh grass in mixed diets for cattle | - **Value of GHG mitigation**: Up to 39% reduction in enteric CH_4_ emissions (Cameron et al., 2018) - **Energy**: Not available - **Productivity and/ or cost**: The costs of any longer-term reductions in milk yields may be outweighed by the benefits of improved farm profitability and reduced GHG emissions (Cameron et al., 2018) - **Abatement cost:** Not available in the covered literature - **Environmental impacts:** Environmentally efficient (Cameron et al., 2018) |
| 3: Livestock performance recording for achieving higher genetic merit | - **Value of GHG mitigation**: Up to 18% reduction in C footprint (case of sheep production farm) (Morgan-Davies et al., 2021) - **Energy**: Not available in the covered literature - **Productivity and/ or cost**: Maintaining farm efficiency and profitability Increased economic margins (by £6/ewe in the case of sheep production)(Morgan-Davies et al., 2021) - **Abatement cost:** Not available in the covered literature - **Environmental impacts:** Environmentally efficient (Morgan-Davies et al., 2021) - **Challenges or trade-offs:** Increased workload within 10% extra labour (Morgan-Davies et al., 2021) |
| D: Nutrient management | |
| 1: Soil amendment (e.g., basalt amendment, biosolids) of croplands | - **Value of GHG mitigation**: Soil organic carbon sequestration increase (Up to 17% for biosolids application and 4 folds increase for basalt amendment), compared to control plant–soil systems without amendment (Kelland et al., 2020; Nicholson et al., 2018) - **Energy**: Reduces the energy demands for milling and the associated carbon emissions penalty from the use of fossil fuels (case of basalt application), compared with conventional treatments, as reported by Kelland et al. (2020) - **Productivity and /or cost**: Crop yield increase, compared to untreated systems (Kelland et al., 2020; Nicholson et al., 2018) - **Abatement cost:** Not available in the covered literature - **Environmental impacts:** Improves soil quality and fertility and increases water infiltration rate (Kelland et al., 2020; Nicholson et al., 2018) |
| 2: Application of nitrification inhibitors (e.g., DMPP, NBPT, DCD, NaClO_3_) in agricultural soils | - **Value of GHG mitigation**: 13 to 60% reduction in N_2_O emissions (Carswell et al., 2019b; Chadwick et al., 2018; Fu et al., 2018; Hargreaves et al., 2021; Wu et al., 2017) - **Energy**: Not available in the covered literature - **Productivity and/ or cost**: Cost effective mitigation measure (Carswell et al., 2019a) - **Abatement cost:** Not available in the covered literature - **Environmental impacts:** Decreases the potential of nitrate leaching and run off ( Carswell et al., 2019a; Hargreaves et al., 2021) - **Challenges or trade-offs:** Potential increase in NH_3_ volatilisation (Wu et al., 2017) |
| 3: Introducing legumes in grasslands and crop rotations | - **Value of GHG mitigation**: Up to 58 % reduction in N_2_O emissions (Carswell et al., 2019b; Costa et al., 2021; Fuchs et al., 2020) - **Energy**: Positive impact on resource use energy (less use of energy for fertilization supply)(Costa et al., 2021) - **Productivity and/ or cost**: Reduction in the costs (e.g., fertilizers) (Carswell et al., 2019b; Costa et al., 2021; Fuchs et al., 2020) - **Abatement cost:** Not available in the covered literature - **Environmental impacts:**  Reduction in marine and terrestrial eutrophication; acidification terrestrial and freshwater; ecotoxicity freshwater and ozone formation (Costa et al., 2021)   Symbiotically fixed nitrogen provides a supply of nitrogen for plants that is more synchronous to plant demand than occasional fertilizer applications (Carswell et al., 2019b; Costa et al., 2021; Fuchs et al., 2020) |
| E: Water table management | |
| Mitigation measure | Implications |
| Raising the water table in peatlands used for agricultural production | - **Value of GHG mitigation**: 0.7% of the total UK GHG emissions (in the case of halving water table depth) (Evans et al., 2021) - **Energy**: There is a need for energy-intensive pumped drainage (Evans et al., 2021) - **Productivity and/ or cost**: The effects of water table depth on crop productivity depends on the crop species (Musarika et al., 2017) - **Environmental impacts:** Improvement in SOC sequestration (Evans et al., 2021) - **Challenges or trade-offs:** Drainage may lead to oxidative loss of peat, land subsidence, increased fire and flood risk (Evans et al., 2021) |

**Table 2** *Summary of the mitigation measures related to offsetting*

| Mitigation measure | Implications |
| --- | --- |
| 1: Cover crops and reduced tillage | - **Value of GHG mitigation**: Up to 25% increase in SOC accumulation; Up to 27% reduction of Great Britain agriculture emissions (through SOC sequestration) (Jordon et al., 2022; Alskaf, 2018) - **Energy**: Reduction in energy use (Glenk et al., 2017) - **Productivity and/ or cost**: Risk of yield reduction in the short, medium term; requires policy intervention including financial incentives for growers during the early stages of the transition from ploughing to conservation agriculture to cover any potential yield reduction (Alskaf, 2018) - **Abatement cost**: Not available in the covered literature - **Environmental impacts:** Benefits to the soil and the environment including reducing runoff, enhancing water retention, and preventing soil erosion (Jordon et al., 2022) - **Challenges or trade-offs**: Weed management and slugs were identified as main challenges for reduced tillage adopters (Alskaf, 2018) |
| 2: Agroforestry systems: Planting trees and/or hedgerows in agricultural soils | - **Value of GHG mitigation**: Up to 2 times higher SOC content (Nworji, 2017)   NH_3_ reduction by 53% in farm emissions (Pagella and Whistance, 2019)   - **Energy**: Biomass energy production instead of non-renewable energy sources (Pagella and Whistance, 2019) - **Productivity and/ or cost**: Maintained or increased productivity   High initial investment cost (Nworji, 2017)  Economic benefits thanks to product diversification (e.g., food, recreation and leisure, foliage, biochar, and Christmas trees…) (Dunn et al., 2021)   - **Abatement cost:** 55 £ (t CO_2_e)^-1^ (O’Neill et al., 2020) - **Environmental impacts:**  Trees can act as wind breaks, contribute to microclimate amelioration, provide shelter for livestock, conservation of soil and water (by slowing the flow of run-off from farms and reducing nitrate leaching as well as soil erosion) (Crous-Duran et al., 2020; Dunn et al., 2021; Jordon et al., 2020; Nworji, 2017; O’Neill et al., 2020; Pagella and Whistance, 2019) - **Challenges or trade-offs**: Technical expertise for establishing agroforestry systems may be needed (Nworji, 2017) |
| 3: Land use change (eg., Conversion of a fallow to arable land or grassland) | - **Value of GHG mitigation**: Up to 4 times higher SOC content (Zhang et al., 2021) - **Energy:** Not available - **Productivity and/ or cost**: More productivity by the conversion of land taken out of production (Baddeley et al., 2017) - **Abatement cost:** Not available in the covered literature - **Environmental impacts:** Increasing the soil aggregate porosity and diffusion (Zhang et al., 2021)   Pollution mitigation, such as carbon sequestration and reduction of nitrate leaching (Baddeley et al., 2017)   - **Challenges or trade-offs:** Slow process to accumulate SOC (Zhang et al., 2021) |

**Table 3** *Summary of the mitigation measures related to bioenergy production*

| Mitigation measure | Implications |
| --- | --- |
| 1: Planting bioenergy crops (namely Willow crops and Miscanthus) | - **Value of GHG mitigation**: Up to 53 MtCO_2_e (considering bioenergy coupled with carbon capture and storage) by 2050, in UK GHG emissions of land use and energy sectors (BEIS 2021) - **Energy**: Bioenergy is viable sustainable alternative to conventional fossil fuel energy source as it improves energy security (with net fossil resource saving of 23,870 Mj) (BEIS 2021; Robertson et al., 2017; Yesufu et al., 2020) - **Productivity and/ or cost**: Cost effective (For instance, lower costs in cultivating Miscanthus than cultivating arable crops) (BEIS 2021; Yesufu et al., 2020) - **Abatement cost:** Not stated in the covered literature. - **Environmental impacts:**  Habitat provisioning and soil and water quality and the climate regulation BEIS 2021; Robertson et al., 2017; Yesufu et al., 2020; Harris et al., 2017) - **Challenges or trade-offs**: Bioenergy crops should be concentrated on less-productive ‘marginal’ land to not cause a conflict between food and bioenergy production on higher-quality soils (Gregory et al., 2018; Ni et al., 2019) |
| 2: Use of agricultural waste (e.g., poultry litter gasification) to produce bioelectricity | - **Value of GHG mitigation**: Around 0.4% of UK’s GHG emissions (Jeswani et al., 2019) - **Energy**: Litter gasification could potentially provide 0.6% of electricity and heat in the UK and save 1.7 Mt of GHG per year (Jeswani et al., 2019) - **Productivity and/ or cost**: High capital costs, the unsubsidised cost of generating heat and electricity from poultry litter is similar to that of natural gas *Combined heat and power* (CHP) but significantly cheaper than from other fossil-fuel alternatives (Jeswani et al., 2019) - **Abatement cost:** 34 £ (t CO_2_e)^-1^ (Jeswani et al., 2019) - **Environmental impacts:** Higher depletion of minerals (3 times higher compared CHP from natural gas) (Jeswani et al., 2019) - **Challenges or trade-offs:** Human toxicity 25% higher compared to CHP from natural gas (Jeswani et al., 2019) |
